# The *Spirit of Transportation* in a Connected World

**DOI:** 10.3201/eid3002.AC3002

**Published:** 2024-02

**Authors:** Nkuchia M. M’ikanatha, Byron Breedlove, David P. Welliver

**Affiliations:** Pennsylvania Department of Health, Harrisburg, Pennsylvania, USA (N.M. M’ikanantha);; Centers for Disease Control and Prevention, Atlanta, Georgia, USA (B. Breedlove);; Clarific Services, Rochester, Minnesota, USA (D.P. Welliver).

**Keywords:** Karl Bitter, The Spirit of Transportation in a Connected World, William H. Gray III 30th Street Station, spread of pathogens, antimicrobial resistance, global connectivity, art and science, about the cover

**Figure:**
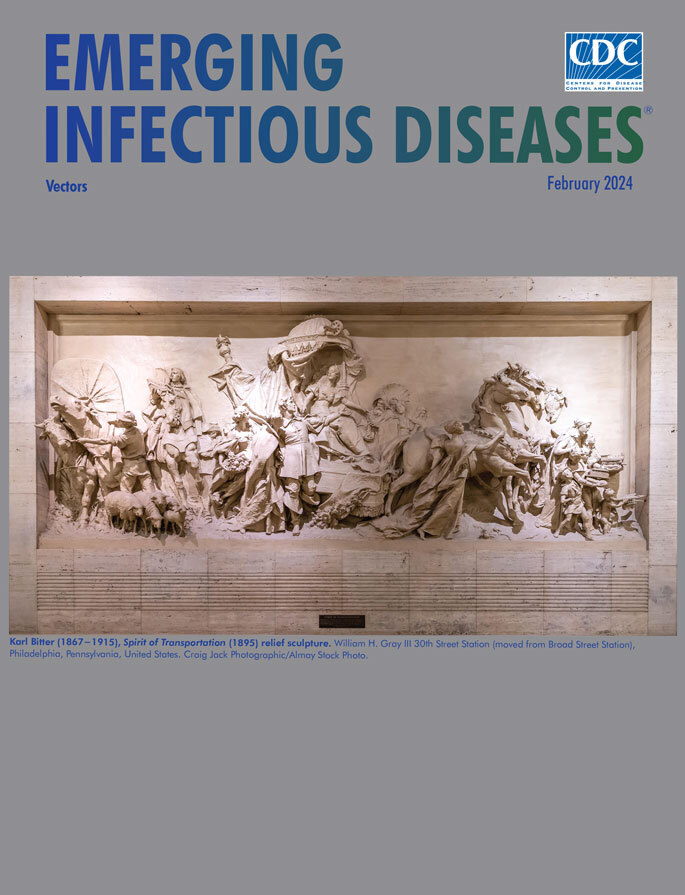
Karl Bitter (1867−1915), *Spirit of Transportation* (1895) relief sculpture. William H. Gray III 30th Street Station (moved from Broad Street Station), Philadelphia, Pennsylvania, United States. Craig Jack Photographic/Almay Stock Photo.

In early 2020, concerns about the spread of SARS-CoV-2 halted all but essential travel, causing bustling seaports, airports, and railroad stations around the world to go quiet. The same scenario played out at the Philadelphia 30th Street Station, recently renamed the William H. Gray III 30th Street Station. That busy train station is the home of *Spirit of Transportation,* an exquisitely detailed 30-foot relief sculpture that has been displayed there since 1933. Completed in 1895 by Karl Bitter, the 3-dimensional work celebrates a triumphant march into modernity, depicted as a procession accompanying a carriage pulled by gallant horses. Leading the way are youths carrying representations of various modes of travel: train, ship, and—carried by the youngest child—a model of a futuristic airship. The Spirit herself, in the middle, is riding on an elaborate carriage trailed by a wagon pulled by uncooperative oxen. As Bitter’s sculpture shows, transportation enables movement of people and cargo, including animals, via various vessels by land, sea, or air. Such connectivity, however, facilitates (usually unintentionally) the spread of disease-causing microbes, including emerging pathogens.

Bitter was born in 1867 in Vienna, Austria, where he studied at various institutions, including the Academy of Painting and Sculpture. He worked as an assistant to the sculptor Joseph Kaffsack, and that experience triggered his interest in architectural decorative art. In 1889, Bitter immigrated to New York, New York, where he was recruited by Richard Morris Hunt, a leading Beaux-Arts architect. They worked on various projects, such as the Administration Building exhibits for the 1893 World’s Fair in Chicago (World’s Columbian Exposition). Other collaborations included decorations for the Biltmore House on the Biltmore Estate near Asheville, North Carolina—a life-size bronze fountain sculpture of a boy with geese is a prominent example.

In later years, Bitter’s work shifted away from naturalism to a modernist tone inspired by Greek and Viennese elements. Notable examples are the 4 allegorical muses on the façade of New York’s Metropolitan Museum of Art along 5th Avenue—the figures were carved from Indiana limestone to represent Sculpture, Painting, Architecture, and Music. Bitter served as the sculpture director for the 1901 Pan-American Exposition in Buffalo, New York, and the 1915 Panama-Pacific Exposition in San Francisco, California. His awards included the silver medal at the 1900 Paris Exposition and the gold medal at the 1904 Saint Louis Exposition. He was also a member of prestigious professional societies, including the American Academy of Arts and Letters.

In his book describing the 1901 Exposition, Professor Kerry S. Grant, former dean of the College of Arts and Sciences at the State University of New York at Buffalo, offers this perspective: “[Bitter] fervently believed that the decorative arts should do more than merely please the senses. They should also convey the purpose of the Exposition.” *Abundance*, a model for the Pulitzer Fountain in front of the Plaza Hotel, at 5th Avenue and 59th Streets in New York, was Bitter’s last sculpture. Bitter died on April 11, 1915, shortly after he was struck by a car as he left the Metropolitan Opera House. He was eulogized by the National Academy of Design as an adopted member honored for “representing American ideals in sculpture.”

*Spirit of Transportation* embodies the human quest to overcome geographic barriers, culminating with the arrival of air travel. Envisaged in the hands of the youngest boy leading the Spirit, that advancement now enables an individual to move from one location on the globe to another with astonishing speed. Before the still lingering coronavirus pandemic COVID-19, the International Civil Aviation Organization estimated that 100,000 commercial flights took off and landed daily worldwide, carrying more than 12 million passengers.

Although rapid transportation has expedited travel around the world, it has also expedited movement of pathogens. Global connectivity through air and other modes of transportation enables the rapid spread of emerging infectious diseases. For instance, airline travel introduced both SARS-CoV and SARS-CoV-2 to various regions of the world, and the recent emergence of Zika virus and its subsequent spread to other regions was fueled by international travel. The movement of humans or animals can lead to transmission of other vectorborne pathogens reported in this month’s issue. For example, the World Health Organization Collaborative Center for Rickettsial Diseases at Marseille, France, documented 32 cases of murine typhus among travelers returning to France from different regions during 2008–2010. S.L. Hills and colleagues reported 6 cases of travel-associated tick-borne encephalitis in the United States during 2010–2020. In addition, transportation of domesticated animals—for example, moving horses infected with the West Nile Virus—can enable emergence or reemergence of vectorborne pathogens. What is more, transportation of companion animals can equally enable the spread of zoonotic pathogens, as in the recent bearded dragon–associated *Salmonella* Vitkin outbreak reported by the Centers for Disease Control and Prevention.

International travel may also accelerate the spread of antimicrobial resistance in foodborne pathogens and drug-resistant sexually transmitted pathogens. Moreover, unvaccinated travelers have been associated with the reintroduction of measles in countries where the disease had previously been eliminated.

Sea transportation is associated with the spread of diseases within and across countries. In 1991, after Latin America had been cholera free for a century, a ship from a cholera-endemic area introduced the disease into Peru, igniting a massive epidemic (1991−1994) that resulted in more than 1 million infections and 9,600 deaths in the Western Hemisphere.

Although modern transportation has, in a sense, dissolved geographic barriers and ushered in globalization, it has inadvertently multiplied opportunities for the spread of pathogens. Measures such as vaccination to prevent typhoid, prophylaxis for malaria, screening of travelers, and isolation of sick passengers can help reduce travel-associated diseases. The Centers for Disease Control and Prevention’s *CDC Yellow Book: Health Information for International Travel* offers practical evidence-based guidelines for making travel safer. In addition, the International Society for Travel Medicine provides a convenient online clinic directory for pretravel and posttravel consultation. Embracing the *Spirit of Transportation* in a connected world means welcoming innovations in transportation—as Bitter’s sculpture illustrates—while simultaneously mitigating, to the extent possible, travel-associated infections.

## References

[1] Angelo KM, Gastañaduy PA, Walker AT, Patel M, Reef S, Lee CV, et al. Spread of measles in Europe and implications for US travelers. Pediatrics. 2019;144:e20190414. 10.1542/peds.2019-0414 31209161 PMC6657509

[2] Biltmore Company. Boy Stealing Geese [cited 2023 April 19] https://www.biltmore.com/blog/parkers-favorites-in-biltmore-house

[3] Guthmann JP. Epidemic cholera in Latin America: spread and routes of transmission. J Trop Med Hyg. 1995;98:419–27. 8544225

[4] Columbia University Digital Library Collection. No. 139–Group for Administration Building—Carl Bitter, Sculptor. Statuary for World’s Columbian Exposition, Chicago [cited 2023 Apr 19]. https://dlc.library.columbia.edu/catalog/cul:2z34tmpj00

[5] European Centre for Disease Prevention and Control. Zika virus disease—annual epidemiological report for 2018 [cited 2023 May 22]. https://www.ecdc.europa.eu/en/publications-data/zika-virus-disease-annual-epidemiological-report-2018 22114980

[6] EverGreene. Karl Bitter’s façade sculptures. Metropolitan Museum of Art, New York, New York [cited 2024 Jan 11]. https://evergreene.com/projects/bitters-facade-sculptures

[7] Findlater A, Bogoch II. Human mobility and the global spread of infectious diseases: a focus on air travel. Trends Parasitol. 2018;34:772–83. 10.1016/j.pt.2018.07.004 30049602 PMC7106444

[8] Grant KS, Seiner WH. The Rainbow City: celebrating light, color, and architecture at the Pan-American Exposition, Buffalo 1901. Buffalo (NY): Canisius College Press; 2001.

[9] Hills SL, Broussard KR, Broyhill JC, Shastry LG, Cossaboom CM, White JL, et al. Tick-borne encephalitis among US travellers, 2010-20. J Travel Med. 2022;29:taab167. 10.1093/jtm/taab167 34741518

[10] Matteelli A, Carosi G. Sexually transmitted diseases in travelers. Clin Infect Dis. 2001;32:1063–7. 10.1086/319607 11264035

[11] Morens DM, Folkers GK, Fauci AS. The challenge of emerging and re-emerging infectious diseases. Nature. 2004;430:242–9. 10.1038/nature02759 15241422 PMC7094993

[12] Smithsonian Archives of American Art. Karl Theodore Francis Bitter papers, 1887–circa 1977 [cited 2024 Jan 11]. https://www.aaa.si.edu/collections/karl-theodore-francis-bitter-papers-8889/biographical-note

[13] New York City Public Art. Pulitzer Fountain, 1914−1916 [cited 2023 Apr 19]. http://www.blueofthesky.com/publicart/works/pulitzer.htm

[14] Olsen SJ, Chang HL, Cheung TYY, Tang AFY, Fisk TL, Ooi SPL, et al. Transmission of the severe acute respiratory syndrome on aircraft. N Engl J Med. 2003;349:2416–22. 10.1056/NEJMoa031349 14681507

[15] Porse CC, Messenger S, Vugia DJ, Jilek W, Salas M, Watt J, et al. Travel-associated Zika cases and threat of local transmission during global outbreak, California, USA. Emerg Infect Dis. 2018;24:1626–32. 10.3201/eid2409.180203 30124194 PMC6106427

[16] Tien V, Punjabi C, Holubar MK. Antimicrobial resistance in sexually transmitted infections. J Travel Med. 2020;27:taz101. 10.1093/jtm/taz101 31840758

[17] Timoney PJ. Infectious diseases and international movement of horses. Equine Infectious Diseases. 2014;544–51.e1.

[18] United Nations. Department of Economic and Social Affairs Sustainable Development. International Civil Aviation Organization (ICAO) [cited 2024 Jan 11]. https://sdgs.un.org/un-system-sdg-implementation/international-civil-aviation-organization-icao-49514

[19] Walter G, Botelho-Nevers E, Socolovschi C, Raoult D, Parola P. Murine typhus in returned travelers: a report of thirty-two cases. Am J Trop Med Hyg. 2012;86:1049–53. 10.4269/ajtmh.2012.11-0794 22665617 PMC3366521

